# MTA1, a Novel ATP Synthase Complex Modulator, Enhances Colon Cancer Liver Metastasis by Driving Mitochondrial Metabolism Reprogramming

**DOI:** 10.1002/advs.202300756

**Published:** 2023-07-13

**Authors:** Ting Wang, Fangzhou Sun, Chunxiao Li, Peng Nan, Yan Song, Xuhao Wan, Hongnan Mo, Jinsong Wang, Yantong Zhou, Yuzheng Guo, Aya Ei Helali, Dongkui Xu, Qimin Zhan, Fei Ma, Haili Qian

**Affiliations:** ^1^ Key Laboratory of Carcinogenesis and Translational Research (Ministry of Education/Beijing) Laboratory of Molecular Oncology Peking University Cancer Hospital & Institute Beijing 100142 China; ^2^ State Key Laboratory of Molecular Oncology National Cancer Center/National Clinical Research Center for Cancer/Cancer Hospital Chinese Academy of Medical Sciences and Peking Union Medical College Beijing 100021 China; ^3^ Department of Medical Oncology National Cancer Center/National Clinical Research Center for Cancer/Cancer Hospital Chinese Academy of Medical Sciences and Peking Union Medical College Beijing 100021 China; ^4^ Laboratory Medicine Center Department of Clinical Laboratory Zhejiang Provincial People's Hospital (Affiliated People's Hospital, Hangzhou Medical College) Hangzhou 310014 China; ^5^ Department of Pathology National Cancer Center/National Clinical Research Center for Cancer/Cancer Hospital Chinese Academy of Medical Sciences and Peking Union Medical College Beijing 100021 China; ^6^ School of Electrical Engineering and Automation Wuhan University Wuhan 430000 China; ^7^ Department of Clinical Oncology Li Ka Shing Faculty of Medicine University of Hong Kong Hong Kong 999077 China; ^8^ Department of VIP National Cancer Center/National Clinical Research Center for Cancer/Cancer Hospital Chinese Academy of Medical Sciences and Peking Union Medical College Beijing 100021 China; ^9^ Peking University International Cancer Institute Peking University Beijing 100191 China; ^10^ Institute of Cancer Research Shenzhen Bay Laboratory, Cancer Institute, Shenzhen Key Laboratory of Gastrointestinal Cancer Translational Research, Peking University Shenzhen Hospital, Shenzhen Peking University‐the Hong Kong University of Science and Technology (PKU‐HKUST) Medical Center Shenzhen 518107 China; ^11^ Research Unit of Molecular Cancer Research Chinese Academy of Medical Sciences Beijing 100021 China; ^12^ Department of Medical Oncology National Cancer Center/National Clinical Research Center for Cancer/Hebei Cancer Hospital Chinese Academy of Medical Sciences Langfang 065001 China

**Keywords:** adenosine triphosphate (ATP), colorectal cancer, mitochondrial glucose metabolism, metastasis‐associated antigen 1 (MTA1), mTOR inhibitors

## Abstract

Liver metastasis is the most fatal event of colon cancer patients. Warburg effect has been long challenged by the fact of upregulated oxidative phosphorylation (OXPHOS), while its mechanism remains unclear. Here, metastasis‐associated antigen 1 (MTA1) is identified as a newly identified adenosine triphosphate (ATP) synthase modulator by interacting with ATP synthase F1 subunit alpha (ATP5A), facilitates colon cancer liver metastasis by driving mitochondrial bioenergetic metabolism reprogramming, enhancing OXPHOS; therefore, modulating ATP synthase activity and downstream mTOR pathways. High‐throughput screening of an anticancer drug shows MTA1 knockout increases the sensitivity of colon cancer to mitochondrial bioenergetic metabolism‐targeted drugs and mTOR inhibitors. Inhibiting ATP5A enhances the sensitivity of liver‐metastasized colon cancer to sirolimus in an MTA1‐dependent manner. The therapeutic effects are verified in xenograft models and clinical cases. This research identifies a new modulator of mitochondrial bioenergetic reprogramming in cancer metastasis and reveals a new mechanism on upregulating mitochondrial OXPHOS as the reversal of Warburg effect in cancer metastasis is orchestrated.

## Graphical abstract

Metastasis‐associated antigen 1 (MTA1), as a newly identified ATP synthase modulator by interacting with ATP synthase F1 subunit alpha, facilitates colon cancer liver metastasis by driving mitochondrial bioenergetic metabolism reprogramming and enhancing OXPHOS; therefore, modulating ATP synthase activity and downstream mTOR pathways. Knockout of MTA1 increases the sensitivity of colon cancer to mitochondrial bioenergetic metabolism‐targeted drugs, mTOR inhibitors.

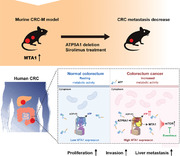

## 1 Introduction

Colon cancer is the third most common and second deadliest malignancy worldwide.^[^ ^1^ ^]^ Metastasis is the leading cause of death among colon cancer patients. The liver is the most common target organ of colon cancer metastasis. Patients with liver metastases have a 5‐year survival rate of 17%, and the median survival of patients with unresectable liver metastases is as short as 13–18 months.^[^ ^2^ ^]^ However, how colon cancer metastasizes to the liver remains unclear, leaving developing therapeutic strategies for this disease challenging and an urgent need for detailed mechanism and targetable molecular hubs.^[^ ^3^ ^]^

Efforts have been made to elucidate the mechanism by which cancer metastasizes to the liver, and in‐depth research has revealed that glucose metabolism reprogramming is one of the key causes of metastatic potential acquisition and metastasis development.^[^ ^4^ ^]^ Alterations in mitochondrial metabolism contribute to tumor progression.^[^ ^5^ ^]^ Warburg effect in tumors, namely increased aerobic glycolysis and decreased oxidative phosphorylation (OXPHOS), was reported to be one of the main driving forces to fuel metastasis. However, this concept has been challenged recently because OXPHOS was demonstrated to be elevated in most cancer cells but not suppressed as expected.^[^ ^6^ ^]^ Mitochondrial respiration and increases in OXPHOS activity are crucial for tumor survival and metastasis.^[^ ^7^ ^]^ However, how mitochondrial glucose metabolism promotes tumor metastasis remains to be elucidated.

As one of the key cellular machines, ATP synthase produces ATP to support all life activities that require energy. Thanks to technical advancements, a large number of studies on the structure and function of ATP synthase under physiological and pathological conditions have been performed.^[^ ^8^ ^]^ Mammalian mitochondrial ATP synthase (MAS) consists of 28 subunits of 17 polypeptides assembled into a membrane‐bound rotor and a cytoplasmic catalytic center linked by a central stalk and a peripheral stalk (PS). The catalytic crown harbors three β subunits, which contain catalytic sites, and three noncatalytic α subunits.^[^ ^9^ ^]^ The structure of ATP synthase is relatively conserved between species, while ATP production efficiency is regulated by a number of mechanisms.^[^ ^10^ ^]^ Mitochondria are referred to as the “energy producing powerhouse” for its role in cellular and cancerous activities.^[^ ^11^ ^]^ OXPHOS and the electron transport chain, the core of mitochondrial functions, are considered the hub therapeutic targets against cancer. Due to the complexity of ATP synthase and its importance in cellular activities, much research on its structural components and functional indications in cancer treatment, which requires rebalancing of energy consumption, is needed.

Metastasis‐associated antigen 1 (MTA1), which was previously reported to be an oncogenic contributor to nucleosome remodeling and component of the deacetylation complex (NuRD), which orchestrates chromatin structure by modifying histones, has been shown to be involved in regulating various biological processes in tumor cells by coupling with Histone deacetylase through its ELM2‐SANT domains,^[^ ^12^ ^]^ promoting tumor cell growth, invasion, and metastasis.^[^ ^13^ ^]^ We previously found the subcellular localization of MTA1 also including cytoplasm.^[^ ^14^ ^]^ The nuclear MTA1 and cytoplastic MTA1 coordinate the phenotypes of colon cancer tissues and cells through different mechanisms, including by acting as an RNA‐binding chromatin‐associated protein to regulate mitosis.^[^ ^15^ ^]^ Here, we demonstrate that MTA1 interacts with the ATP synthase complex in mitochondria to facilitate the growth and liver metastasis of colon cancer by driving mitochondrial glucose metabolism reprogramming.

To determine the potential clinical relevance of MTA1‐mediated ATP production via mitochondrial glucose metabolism, we assessed its association with drug sensitivity through the core ATP synthase component ATP synthase F1 subunit alpha (ATP5A) and downstream targets of the mTOR pathway by screening an anticancer drug library.

## 2 Results

### 2.1 Combined Transcriptomic and Metabolomic Analysis Reveals the Role of MTA1 in OXPHOS

Mitochondrial OXPHOS metabolic reprogramming is essential for facilitating metastasis.^[^ ^16^ ^]^ We analyzed the intensity of mitochondrial metabolism in colorectal cancer (CRC) liver metastasis by Gene set enrichment analysis (GSEA) and found that expression of the genes upregulated in liver metastasis compared with primary CRC highlighted the processes of glucose import, glucose metabolism process, and ATP metabolism (Figure S1, Supporting Information). Co‐expression analysis combined with GO functional enrichment analysis can be used to reliably predict the potential biological functions of target genes.^[^ ^15b^ ^]^ GO functional enrichment analysis of genes co‐expressed with MTA1 (absolute value of Spearman correlation coefficient greater than 0.3) according to pan‐cancer transcriptome data from the TCGA was performed. Consistent with the known functions of MTA1, we found that genes co‐expressed with MTA1 were mainly associated with RNA processing, the response to DNA damage, macromolecule biosynthesis and transportation, chromosome organization, histone modification, and cell cycle‐related pathways (Figure S2, Supporting Information); these findings prove the reliability of this analytical method. Besides, cell metabolism regulation, which involves a subset of genes connecting mitochondrion organization and localization, protein transport to mitochondrion, and mitochondria disassembly, was highlighted among the enriched terms. This finding expands our knowledge of the role of MTA1 in the mitochondrial energy metabolism in cancers.

**Figure 1:**
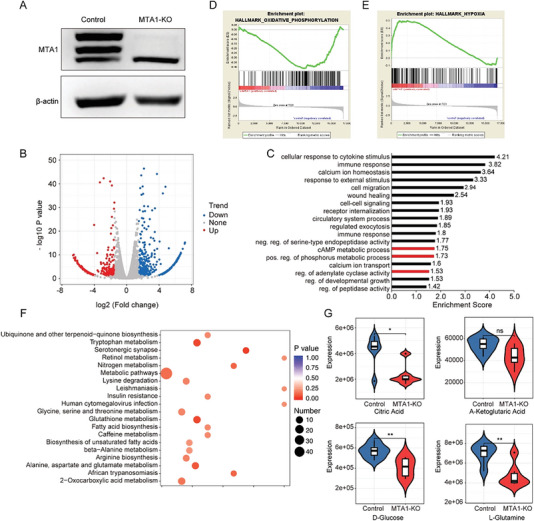
Combined transcriptomics and metabolomics analysis reveals that MTA1 plays a role in glucose metabolism. A) Western blotting was performed to verify that MTA1 was knocked out in HCT116 cells. Representative results from three independent experiments with similar findings are shown. B) Volcano plot showing the differentially expressed genes in MTA1‐KO HCT116 cells. C) Functional enrichment analysis of the downregulated genes using the DAVID 6.8 database. D,E) GSEA of the differentially expressed genes in MTA1‐KO HCT116 cells. F) KEGG pathway analysis of 96 differentially expressed metabolites in MTA1‐KO HCT116 cells. G) Violin plots of the differentially expressed metabolites citric acid (top left), α‐ketoglutaric acid (top right), glucose (bottom left), and L‐glutamine (bottom right), *n* = 6 replicate/group. The values are the mean ± SD (**p < 0.01, *p < 0.05; ns: not significant).

We hypothesized that MTA1 participates in CRC metabolism to modulate its liver metastasis. We constructed an MTA1 knockout (MTA1‐KO) HCT116 cell line by clustered regularly interspaced short palindromic repeats/CRISPR‐associated protein 9 (CRISPR/Cas9) and assessed changes in gene expression by RNA sequencing (**Figure** **1**A). Global gene expression profiling revealed that the expression of 834 protein‐coding genes, 307 of which were downregulated and 527 of which were upregulated, was specifically altered by MTA1‐KO (Figure 1B), supporting the idea that MTA1 is an extensive regulator of gene expression in CRC cells. Interestingly, functional enrichment analysis of the upregulated genes showed that they were mainly involved in organ development‐ and cell motility‐related functions that were previously been reported to be involved in CRC. However, the downregulated genes were enriched in several metabolic pathways (Figure 1C), including the cAMP metabolic process, the phosphorus metabolic process, and regulation of adenylate cyclase activity.GSEA revealed that the expression of genes related to OXPHOS was decreased in MTA1‐KO HCT116 cells (Figure 1D,E), whereas the expression of genes related to hypoxia was increased. Thus, we suspected that mitochondrial OXPHOS was inhibited in MTA1‐KO HCT116 cells, resulting in the development of hypoxic stress.

The most intuitive indicator of gene‐mediated regulation of metabolism is an alteration in metabolite levels in tumor cells. To examine whether MTA1 regulates metabolism and determine its precise localization, we performed metabolomics analysis using liquid chromatography with tandem mass spectrometry (LC–MS/MS). Differential expression analysis revealed that the levels of 94 metabolites were significantly altered, with the levels of 29 metabolites being increased and the levels of 65 metabolites being decreased (fold change < 0.7 or fold change > 1.5) in MTA1‐KO HCT116 versus control cells (Figure S3A,B, Supporting Information). The downregulated metabolites in MTA1‐KO cells were enriched in metabolites of ubiquinone and other terpenoid‐quinone biosynthesis; glycine, serine, and threonine metabolism; glutathione metabolism, and the fatty acid biosynthesis pathway (Figure 1F,G), among which, intermediate metabolites of the tricarboxylic acid cycle, glucose, and glutamine metabolism pathways were related to mitochondrial OXPHOS. Collectively, these data indicate that MTA1 is involved in metabolism regulation in CRC.

### 2.2 MTA1 Enhances the Bioenergetic Activity of Cancer Cells

**Figure 2:**
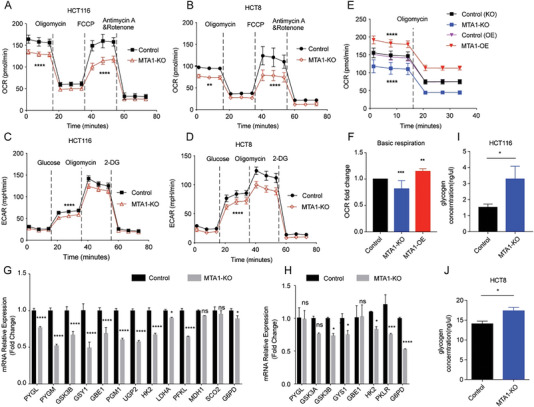
MTA1 enhances global bioenergetic activity. A,B) OCR trace of MTA1‐KO and control HCT116 colon cancer cells (A) and HCT8 (B). The values are the mean ± SD (****p < 0.0001, **p < 0.01), two‐way ANOVA, Sidak's multiple comparisons test. C,D) ECAR trace of MTA1‐KO and control HCT116 (C) and HCT8 (D) cells. The values are the mean ± SD (****p < 0.0001), two‐way ANOVA, Sidak's multiple comparisons test. E,F) Basic respiration of MTA1‐KO and MTA1‐OE HCT116 cells (E) and statistical analysis (F). The values are the mean ± SD (****p < 0.0001, ***p < 0.001, and **p<0.01), *n* = 3 replicate/group, two‐way ANOVA, Tukey's multiple comparisons test. G) The mRNA levels of PYGL, PYGM, GSK3B, GYS1, GBE1, PGM1, UGP2, HK2, LDHA, PFKL, MDH1, SCO2, and G6PD in MTA1‐KO and control HCT116 cells were examined by real‐time PCR. The values are the mean ± SD (****p < 0.0001, *p < 0.05; ns, not significant), Student's *t*‐test, *n* = 3 replicate/group. H) The mRNA levels of PYGL, GSK3A, GSK3B, GYS1, GBE1, HK2, PKLR, and G6PD in MTA1‐KO and control HCT8 cells were examined by real‐time PCR. The values are the mean ± SD (****p < 0.0001, ***p < 0.001, *p < 0.05; ns, not significant), Student's *t*‐test, *n* = 3 replicate/group. I,J) The glycogen level in MTA1‐KO and control HCT116 (I) and HCT8 (J) cells. The values are the mean ± SD (*p < 0.05), Student's *t*‐test, *n* = 3 replicate/group.

To further confirm the biological effects of MTA1‐mediated regulation of mitochondrial OXPHOS in CRC, we assessed related phenotypes by altering MTA1 levels in HCT116 and HCT8 CRC cells. First, we examined the changes in the OCR and ECAR in MTA1‐KO or MTA1‐overexpressing (MTA‐OE) HCT116 cells to assess the balance of mitochondrial activity and glycolysis. We found minimal changes in glycolysis but a drastic reduction in mitochondrial respiration in both cell lines when MTA1 was depleted (**Figure** **2**A–D). Overexpression of MTA1 contributed to an increase in mitochondrial respiration in HCT116 cells (Figure 2E,F).

To verify whether MTA1 modulates energy metabolism pathways by affecting transcription, we measured the transcript levels of energy metabolism‐related genes in CRC cells lacking MTA1. Real‐time PCR suggested that in CRC cells, MTA1 altered the mRNA levels of genes involved in metabolic processes, such as the glycolysis‐related genes hexokinase 2 (HK2), lactate dehydrogenase A (LDHA), phosphofructokinase, liver type (PFKL), pyruvate kinase L/R (PKLR); the pentose phosphate pathway‐related gene glucose‐6‐phosphate dehydrogenase (G6PD); and the glycogen synthesis and breakdown‐related genes glycogen phosphorylase L (PYGL), glycogen phosphorylase, muscle associated (PYGM), glycogen synthase kinase 3 beta (GSK3B), glycogen synthase kinase 3 alpha (GSK3A), glycogen synthase 1 (GYS1), 1,4‐alpha‐glucan branching enzyme 1 (GBE1), phosphoglucomutase 1 (PGM1) and UDP‐glucose pyrophosphorylase 2 (UGP2) (Figure 2G,H). However, MTA1 had almost no effect on the mRNA levels of the mitochondrial respiration‐related genes malate dehydrogenase 1 (MDH1) and synthesis of cytochrome C oxidase 2 (SCO2). Regarding glycogen mobilization, metabolomic analysis revealed that the expression of uridine diphosphate‐glucose (UDP‐G), a glycogen synthesis precursor, was downregulated in MTA1‐KO HCT116 cells (Figure S3C, Supporting Information) and that glycogen accumulated in MTA1‐KO HCT116 and HCT8 cells (Figure 2I,J). We also observed that the mRNA levels of the glycogen breakdown‐related genes PYGM, PYGL, GSK3A and GSK3B (Figure 2G,H) were reduced in in MTA1‐KO cells, indicating that MTA1 may also promote the mobilization of glycogen in CRC cells.

### 2.3 MTA1 Facilitates Mitochondrial Metabolism Reprogramming in CRC Cells

**Figure 3:**
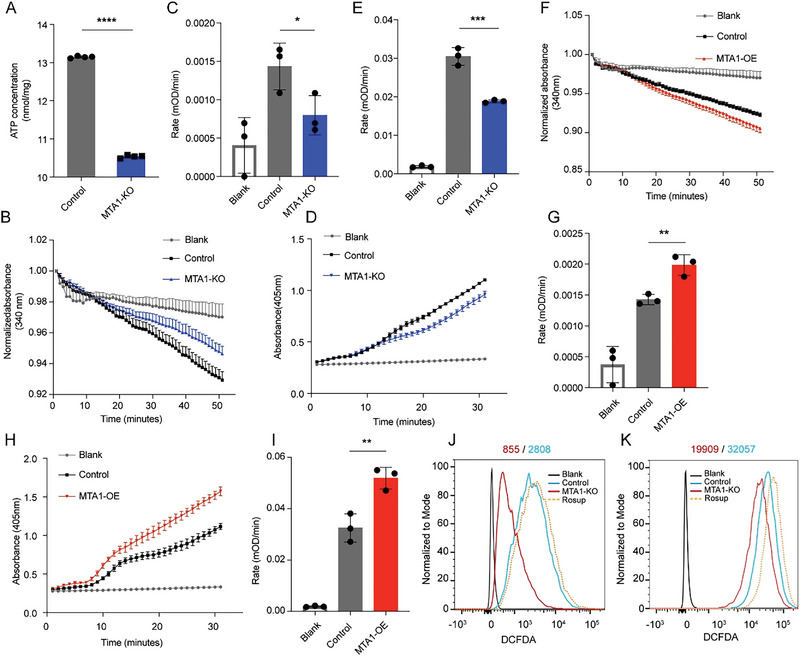
MTA1 facilitates mitochondrial metabolism reprogramming in CRC cells. A) The ATP level in MTA1‐KO and control HCT116 cells. The values are the mean ± SD (****p < 0.0001), Student's *t*‐test, *n* = 4 replicate/group. B,C) The activity of ATP synthase was measured as the molar conversion of NADH to NAD+ and is presented as the decrease in absorbance at 340 nm in MTA1‐KO and control HCT116 cells versus their control cells. The values are the mean ± SEM (B). The rate of decrease in absorbance at 340 nm over time in MTA1‐KO and control HCT116 cells (C). The values are the mean ± SD (*p < 0.05), Student's *t*‐test, *n* = 3 replicate/group. D,E) The level of ATP synthase was measured as the percentage of alkaline phosphatase activity and is presented as the increase in absorbance at 405 nm in MTA1‐KO and control HCT116 cells (D). The rate of increase in absorbance at 405 nm per min in MTA1‐KO and control cells (E). The values are the mean ± SD (***p < 0.001), Student's *t*‐test, *n* = 3 replicate/group. F,G) The activity of ATP synthase was measured as the decrease in absorbance at 340 nm in MTA1‐OE and control HCT116 cells (F). The rate of decrease in absorbance at 340 nm over time in MTA1‐OE and control HCT116 cells (G). The values are the mean ± SD (**p < 0.01), Student's *t*‐test, *n* = 3 replicate/group. H,I) The level of ATP synthase was measured as the increase in absorbance at 405 nm in MTA1‐OE and control HCT116 cells (H). The rate of increase in absorbance at 405 nm per min in MTA1‐OE and control HCT116 cells (I). The values are the mean ± SD (**p < 0.01), Student's *t*‐test, *n* = 3 replicate/group. J,K) DCF intensity in MTA1‐KO and control HCT116 (J) and HCT8 (K) cells. Rosup (50 µg mL^−1^) was used as a positive control in the ROS assay.

Given that bioenergetic activity was decreased in MTA1‐KO CRC cells, we further evaluated mitochondrial energy production in these cells. First, the ATP level in CRC cell lines was determined. We found that the ATP level in CRC cells was decreased after MTA1‐KO (**Figure** **3**A). As ATP is mainly produced by ATP synthase in cells, we then assessed the specific activity of ATP synthase, which may be affected by MTA1. Indeed, MTA1‐KO significantly decreased the activity of ATP synthase (Figure 3B–E), while overexpression of MTA1 increased ATP synthase activity (Figure 3F–I). Reactive oxygen species (ROS), which are thought to act as small signaling molecules even at low levels, are mainly produced as byproducts of OXPHOS and accompanied by ATP synthesis.^[^ ^17^ ^]^ ROS enhancement leads to cellular signaling cascades activation and accumulation of oncogenic mutations. We assessed the influence of MTA1 on ROS production and found that ROS levels were decreased in MTA1‐KO HCT116 and MTA1‐KO HCT8 cells (Figure 3J,K). These results indicated that MTA1 facilitates mitochondrial ATP production in colon cancer cells.

### 2.4 MTA1 Directly Interacts With ATP5A

**Figure 4:**
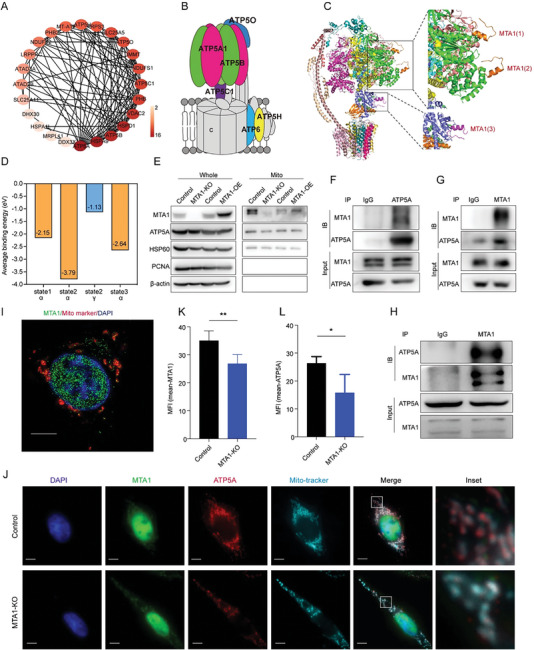
MTA1 interacts with ATP5A. A) Interaction network of 24 mitochondria‐associated proteins that bind MTA1 identified by LC–MS/MS. The shade of color indicates the number of proteins interacting with that protein. B) Schematic of the distribution of MTA1‐binding proteins, which are highlighted with different colors on ATP synthase. C) Simulation of the interaction between amino acids 670‐695 of MTA1 and ATP synthase in state 2. Binding sites are indicated by black boxes and magnified. MTA1 peptides are shown in orange (top) and pink (bottom). The ATP synthase *α* subunit is shown in green. The ATP synthase *γ* subunit is shown in purple. D) Average binding energy of the interaction between MTA1 peptides and ATP synthase subunits determined by simulated electron diffraction patterns (eV). E) Western blot analysis of MTA1 and ATP5A levels in mitochondria in MTA1‐KO cells and MTA1‐OE cells. F,G) Co‐IPs of MTA1 (F) and ATP5A (G) in HCT116 cells. H) Analysis of the binding between MTA1 and ATP5A in HCT8 cells by Co‐IP. I,J) The colocalization of MTA1 and ATP5A in mitochondria in HCT116 parent cells (I) and MTA1‐KO HCT116 cells (J) was visualized by immunofluorescence. Scale bar: 5 µm. K,L) Quantification of immunofluorescence. The values are the mean ± SD (**p < 0.01, *p < 0.05), Student's *t*‐test, *n* = 4 replicate/group.

Previously, we reported that MTA1 is localized in the cytoplasm in cancer cells,^[^ ^14^ ^]^ but its detailed functions remain to be explored. To determine whether MTA1 interacts with glucose metabolism‐related proteins, we identified MTA1‐binding proteins using Co‐immunoprecipitation (Co‐IP) followed by LC–MS/MS‐based protein identification. Rabbit‐ and mouse‐derived MTA1 antibodies captured 27 mitochondria‐associated proteins (Table S1, Supporting Information), accounting for 20% of all captured MTA1‐binding proteins, and 24 proteins among the 27 mitochondria‐associated proteins showed an interaction network according to STRING database (**Figure** **4**A). Most of these proteins are components of ATP production‐related mitochondrial electron transport chain complexes and six are ATP synthase subunits. These proteins are shown in color in the schematic (Figure 4B). From the above results, we hypothesized that MTA1 influences mitochondrial functions in CRC cells by directly interacting with glucose metabolism complexes in mitochondria.

We simulated the potential interaction between MTA1 and the candidate MTA1‐binding mitochondrial complex components by MOPAC2016 to computationally analyze the physical interaction between molecules.^[^ ^18^ ^]^ ATP synthase has three main rotational states, that is, state 1, state 2, and state 3, which result from the threefold symmetry of its catalytic region^[^ ^19^ ^]^ and are related to its ATP production ability. The simulation showed a strong binding affinity between the MTA1 peptide (670‐695 aa) and ATP synthase in state 2 and revealed three potential binding sites, two of which are on ATP synthase subunit α and one of which is on ATP synthase subunit γ (Figure 4C; Figure S4A,B, Supporting Information); however, there was no interaction between the MTA1 peptide and ATP synthase subunit β (Figure S4C, Supporting Information). The interaction between the MTA1 peptide and ATP synthase in state 2 was stronger than that between the MTA1 peptide and ATP synthase in the other two states (Figure 4D). To verify this interaction, we isolated mitochondria from HCT116 cells and extracted mitochondrial protein to verify the localization and level of MTA1 in mitochondria. We found that MTA1 expression was decreased in mitochondria in MTA1‐KO cells and that ATP5A expression was downregulated and MTA1 was overexpressed in mitochondria in MTA1‐OE cells (Figure 4E); these findings confirm that MTA1 is localized in mitochondria. Moreover, consistent with the computational results, Co‐IP confirmed MTA1 interaction with ATP5A in HCT116 (Figure 4F,G) and HCT8 cells (Figure 4H). In line with this, immunofluorescence localization analysis showed that apart from the typical nuclear distribution, cytoplasmic MTA1 was localized in mitochondria (Figure 4I) and colocalized with ATP5A in HCT116 cells (Figure 4J). The fluorescence intensity of ATP5A was significantly reduced in MTA1‐KO HCT116 cells (Figure 4J–L). The colocalization of MTA1 and ATP5A was also verified in mouse derived cancer cell line CT26 and other cancer cell lines, including MCF7, MDA‐MB‐231, and HeLa cells (Figure S4D,E, Supporting Information). Further, we measured the mitochondrial membrane potential (MMP) but found no difference between the MTA1‐KO and control cells (Figure S4F,G, Supporting Information). Collectively, these results indicated that MTA1 interacts with ATP5A in mitochondria to facilitate the production of ATP and the accumulation of ROS.

### 2.5 MTA1 Promotes the Metastatic Behavior of CRC by Interfering With ATP5A Function

**Figure 5:**
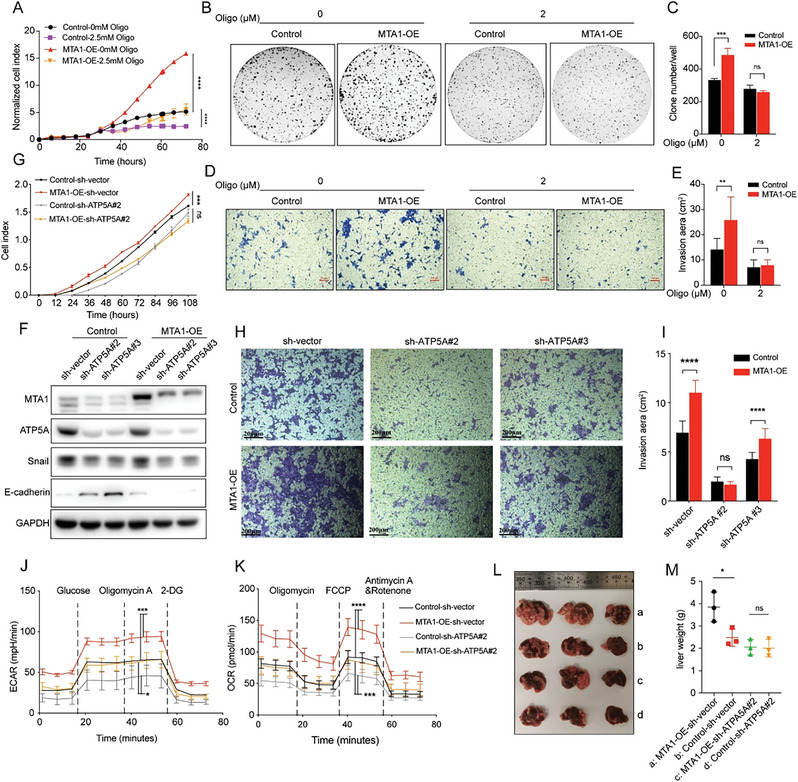
MTA1 promotes the malignant phenotype of CRC via binding with ATP5A. A) The proliferation of MTA1‐OE and control HCT116 cells treated with 2.5 mm oligomycin. The values are the mean ± SD (****p < 0.0001), two‐way ANOVA, Tukey's multiple comparisons test, *n* = 2 replicate per group. B,C) The colony formation ability of MTA1‐OE and control HCT116 cells treated with 2 µm oligomycin for 10 days. The values are the mean ± SD (***p < 0.001; ns, not significant), two‐way ANOVA, Sidak's multiple comparisons test, *n* = 3 replicate/group. D,E) The invasion of MTA1‐OE and control HCT116 cells treated with 2 µm oligomycin for 48 h. The values are the mean ± SD (**p < 0.01; ns, not significant), two‐way ANOVA, Sidak's multiple comparisons test, *n* = 3 replicate/group. Scale bar: 100 µm. F) Western blotting was performed to verify that ATP5A was knocked down in MTA1‐OE and control HCT116 cells, and the expression of metastasis related markers in MTA1‐OE/ATP5A‐KD HCT116 cells. G–I) The proliferation (G) and invasion (H,I) of MTA1‐OE/ATP5A‐KD and control HCT116 cells. The cells were incubated in transwell plates for 48 h. The values are the mean ± SD (****p < 0.0001, ***p < 0.001; ns, not significant), two‐way ANOVA, Tukey's multiple comparisons test, *n* = 3 replicate/group. Scale bar: 200 µm. J,K) The ECAR (J) and OCR (K) of MTA1‐OE/ATP5A‐KD and control HCT116 cells. The values are the mean ± SD (****p < 0.0001, ***p<0.001, and *p<0.05), two‐way ANOVA, Tukey's multiple comparisons test. L) Representative image of the liver metastatic burden in BALB/c‐nu/nu mice injected with MTA1‐OE/ATP5A‐KD and control cells via the spleen tail. The group names are shown in (M); *n* = 3 mice/group. M) The liver weight of mice with liver metastasis shown in (L). The values are the mean ± SD (*p < 0.05; ns, not significant), one‐way ANOVA, Tukey's multiple comparisons test.

Consistent with previous reports, MTA1 was found to promote the survival and invasiveness of CRC cells (Figure S5A–C, Supporting Information). To determine whether MTA1 promotes the metastatic behavior of CRC cells by driving mitochondrial glucose metabolism reprogramming, we treated MTA1‐OE cells with the ATP synthase inhibitor oligomycin A. Oligomycin A treatment significantly abolished the increase in the proliferation, colony formation, and invasiveness of HCT116 cells induced by MTA1 overexpression (**Figure** **5**A–E), suggesting that MTA1 may promote the malignant behavior of CRC cells by enhancing ATP synthase function. Subsequently, we knocked down ATP5A in MTA1‐OE HCT116 cells, finding that metastasis marker E‐cadherin was downregulated in MTA1‐OE cells and upregulated in MTA1 control and ATP5A knockdown cells (Figure 5F). We observed that the increase in cell survival and invasiveness induced by MTA1 overexpression was marked abolished following loss of ATP5A (Figure 5G–I). In addition, the ability of MTA1 overexpression to promote mitochondrial OXPHOS and glycolysis in CRC cells was abolished when ATP5A was knocked down (Figure 5J,K). Further, we established a mouse model of liver metastasis by injecting mice with CT26 and HCT116 cells via the spleen tail. Consistently, liver metastasis was significantly reduced by MTA1 knockdown and greatly increased by MTA1 overexpression (Figure S5D–G, Supporting Information). We observed that the enhancement of liver metastasis was significantly abolished in metastasis model mice injected with MTA1‐OE/ATP5A knockdown (ATP5A‐KD) HCT116 cells compared with those injected with MTA1‐OE cells (Figure 5L,M). Thus, both in vitro and in vivo results demonstrated that MTA1 promotes the proliferation and invasiveness of CRC cells by regulating ATP5A function.

### 2.6 Depletion of MTA1 Increases the Sensitivity of Metastatic CRC to Rapamycin Through the mTOR Pathway

**Figure 6:**
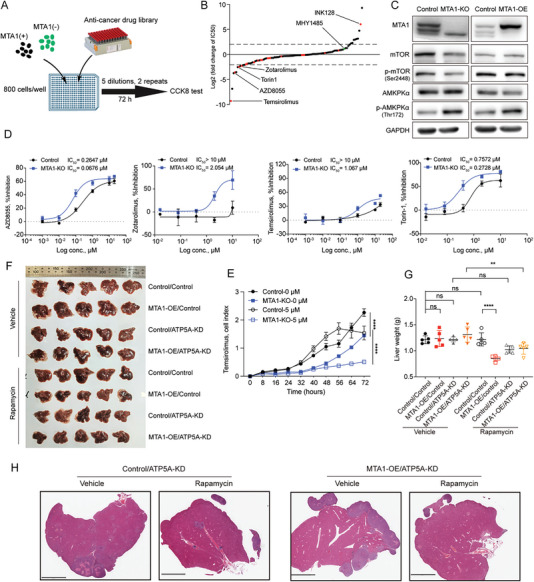
MTA1 increases the efficacy of mTOR inhibitors both in vitro and in vivo. A) Schematic of the experiment. MTA1‐KO and control HCT116 cells were seeded in 384‐well plates (800 cells per well) 16 h before the experiment, and the cells were treated with 237 metabolism‐related anticancer drugs for 72 h (five dilutions per drug, two replicates). Cell viability was assessed by the CCK8 assay. B) Fold change in the IC_50_ of metabolism‐related anticancer drugs in MTA1‐KO cells versus control cells. The red dots are mTOR inhibitors, and the green dots are mTOR activators. C) Western blot analysis of the levels of MTA1, mTOR, p‐mTOR, AMPKα, and p‐AMPKα in MTA1‐KO cells, MTA1‐OE cells, and control HCT116 cells. D) IC_50_ curves of the mTOR inhibitors AZD8055, temsirolimus, zotarolimus, and Torin‐1 in MTA1‐KO and control HCT116 cells for individual pharmacological validation. The values are the mean ± SD. E) The viability of MTA1‐KO and control HCT116 cells treated with 5 µm temsirolimus. The values are the mean ± SD (****p < 0.0001), two‐way ANOVA, and Tukey's multiple comparisons test. F) Image of liver metastatic burden in the eight groups of BALB/c‐nu/nu mice injected with MTA1‐OE/ATP5A‐KD via the spleen tail. The mice were injected intraperitoneally with 100 µL of 8.8 mg mL^−1^ rapamycin every other day 2 weeks after spleen‐tail injection; *n* = 5 mice per group. G) The liver weight of mice with liver metastasis shown in (F). The values are the mean ± SD (****p < 0.0001, **p < 0.01; ns, not significant), one‐way ANOVA, and Tukey's multiple comparisons test. H) Representative HE staining images of the liver tumors of the mice in (F). Scale bar: 3 mm.

MAS is indispensable for cancer behavior. The modulatory effect of MTA1 on mitochondrial glucose metabolism may indicate its value for cancer treatment. To explore the effect of MTA1‐mediated MAS function on the sensitivity of cancer to clinically available anticancer drugs which may be influenced by MTA1‐mediated ATP synthase complex activity, we performed high‐throughput screening of 237 clinically validated or preclinically‐tested metabolism‐related anticancer drugs using MTA1‐KO and control cells (**Figure** **6**A). The targets of these 273 bioactive small molecule compounds are related to carbohydrate, lipid, amino acid, and nucleotide metabolism in cancer cells. According to the changes in the IC_50_ of the drugs (Table S2, Supporting Information), mTOR inhibitors were more cytotoxic to MTA1‐KO cells than the other drugs (Figure 6B). AMP‐activated protein kinase （AMPK cascade is the main energy‐sensing pathway downstream of mitochondrial energic reprogramming.^[^ ^20^ ^]^ The results confirmed that MTA1 exerted its effect through ATP5A and that in which the AMP‐activated protein kinase (AMPK) and mTOR pathways are essential for this effect. We also confirmed that mTOR and p‐mTOR levels were decreased in MTA1‐KO cells and increased in MTA1‐OE cells (Figure 6C). mTOR is a signaling molecule involved in glucose metabolism and a therapeutic target for renal cell carcinoma and breast cancer.^[^ ^21^ ^]^ Indeed, pharmacological validation revealed that MTA1‐KO cells were more sensitive to AZD8055, temsirolimus, zotarolimus, and torin1 (Figure 6D). Further, we observed that the proliferation of MTA1‐KO cells was significantly reduced compared with the proliferation of control cells (Figure 6E). We established a colon cancer liver metastasis model and treated the mice with the mTOR inhibitor rapamycin (Figure S6, Supporting Information). The sensitivity of the tumors to rapamycin was unchanged after knockdown of ATP5A alone (Figure 6F,G, group 3 versus group 7; Figure 6H, top). However, mice bearing tumors with MTA1 overexpression and subsequent knockdown of ATP5A were more sensitive to the treatment and displayed reduced metastasis (Figure 6G, group 4 versus group 8, and Figure 6H, bottom), demonstrating that inhibition of ATP5A enhances the sensitivity of colon cancer liver metastases to the mTOR inhibitor rapamycin and that this process is MTA1 dependent.

### 2.7 MTA1 and ATP5A Levels are Correlated With Sensitivity to Sirolimus in CRC Patients

**Figure 7:**
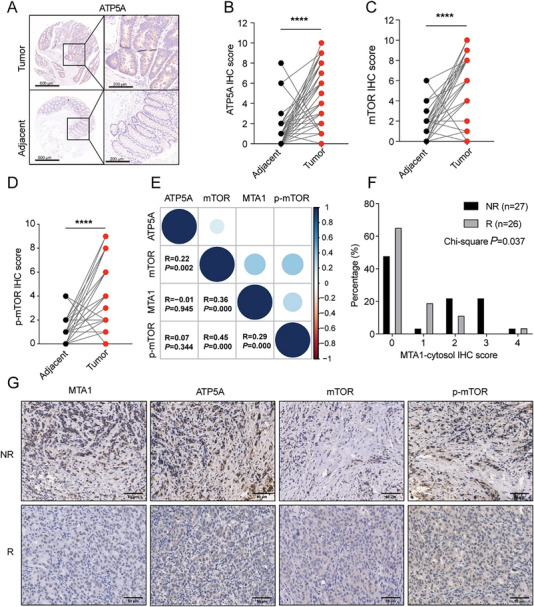
MTA1 and ATP5A levels are correlated with sensitivity to sirolimus in CRC patients. A) Representative images of IHC staining for ATP5A in human tumors and adjacent tissues on 180 CRC tissue arrays. The sample dots (left) were magnified (right) three times. Scale bar: 600 µm. B–D) ATP5A (B), mTOR (C), and p‐mTOR (D) IHC staining scores in 180 paired CRC tissues (****p < 0.0001), Wilcoxon matched‐pairs signed rank test. E) Correlation plot showing the Spearman correlation between MTA1 levels and ATP5A levels, mTOR staining, and p‐mTOR staining in 180 paired CRC tissues (*R* = 0.23, *p* = 0.002), Spearman's rank order correlation coefficient. F) Histogram of cytoplasmic MTA1 protein levels in 58 sirolimus‐ or everolimus‐treated responder and nonresponder breast cancer patients. The Chi‐square test was used to compare the IHC staining score between the above two groups. *p* < 0.05 was considered significant. G) Representative images of IHC staining for MTA1, ATP5A, mTOR, and p‐mTOR proteins in 58 sirolimus‐ or everolimus‐treated responder and nonresponder breast cancer patients. Scale bar: 50 µm.

To verify the clinical relevance of MTA1 and ATP5A expression in tumors from clinical patients, we analyzed the expression of cytoplasmic MTA1, ATP5A, mTOR, and p‐mTOR in tissues from colon cancer patients. IHC staining of tissue arrays containing 180 colon cancer samples revealed that the expression levels of ATP5A, mTOR, and p‐mTOR in tumor tissues were significantly higher than those in paired adjacent tissues (**Figure** **7**A–D). The level of MTA1 was positively correlated with the levels of ATP5A, mTOR, and p‐mTOR (Figure 7E). Subsequently, we obtained paraffin sections from 58 breast cancer patients who received endocrine therapy and trastuzumab combined with sirolimus or everolimus, an mTOR inhibitor approved for the treatment of metastatic renal cell carcinoma and breast cancer patients,^[^ ^21a^ ^]^ and performed IHC staining to analyze the relationship between the cytoplasmic level of MTA1 and the response to mTOR inhibitors. We found that the cytoplasmic level of MTA1 was significantly negatively correlated with the response of breast cancer patients to mTOR inhibitors (Figure 7F,G).

## 3 Discussion

Metabolic reprogramming has been proposed as a hallmark of cancer.^[^ ^22^ ^]^ Macromolecular synthesis is maintained in tumor cells through the reprogramming of energy metabolism, supporting proliferation or metastasis.^[^ ^23^ ^]^ In colon cancer, energy metabolism reprogramming is associated with tumor proliferation, invasion, metastasis, drug resistance, and immune escape.^[^ ^24^ ^]^ Mitochondria are the main suppliers of energy and are responsible for the balance between glycolysis and OXPHOS. MAS, also known as complex V, is the key enzyme for OXPHOS in mitochondria. Our results suggest that mitochondrial MTA1 interacts with ATP5A, the α subunit of MAS, to promote ATP production and facilitate CRC cell proliferation and invasion, further contributing to colon cancer metastasis.

Tumors critically rely on mitochondrial bioenergetic supply. “Warburg effect” is widely accepted as the fundamental theory for glucose metabolism reprogramming in diseases, especially in cancer, in which upregulated aerobic glycolysis and decreased OXPHOS was expected and considered to be a consequence of mitochondrial dysfunction.^[^ ^25^ ^]^ “Warburg effect” fuels cancer metastasis through metabolism reprogramming.^[^ ^26^ ^]^ However, more and more evidence shows that mitochondrial dysfunction is not necessary for tumorigenesis and metastasis as indicated by “Warburg effect;” instead, it is most often overactivated in terms of ATP production by glucose OXPHOS.^[^ ^25a^ ^]^ Our results that MTA1 upregulated mitochondrial glucose OXPHOS during cancer metastasis shed new light on the mechanism of energy balance by “Warburg effect.” Increased OXPHOS in cancer not only provides the high demand of energy for direct expansion but also links the signal crosstalk with tumor microenvironment through byproduct production, such as ROS, which is a pleiotropic physiological signaling agent.^[^ ^27^ ^]^

Mitochondria are organelles with a dynamic fusion and division cycle and a double‐membrane structure and are the main organelles involved in aerobic respiration of cells.^[^ ^28^ ^]^ Respiratory chain complex I, complex II, complex III, complex IV, and complex V are key components for ATP production and are embedded in the inner membrane of mitochondria.^[^ ^29^ ^]^ In this study, we first found that MTA1 is localized in mitochondria. Through Co‐IP‐mass spectrometry, we identified 27 mitochondria‐related proteins that interact with MTA1, including NADH dehydrogenase (ubiquinone) Fe‐S protein 1 (NDUFS1), NADH dehydrogenase (ubiquinone) flavoprotein 2 (NDUFV2) of complex I, ATP5A, ATP synthase F1 subunit beta (ATP5B), ATP synthase F1 subunit gamma (ATP5C), ATP synthase peripheral stalk subunit D (ATP5H) of complex V, and key components of the mitochondrial contact site and cristae organizing system (MICOS), indicating that MTA1 is physically associated with mitochondria. Among these proteins, MTA1‐binding ATP synthase subunits were the most abundant. Further experiments confirmed that the interaction between MTA1 and ATP5A is essential for the regulation of ATP synthase function. Based on the results in this study, MTA1 promotes an increase in the production of ATP by ATP synthase. On the mesoscale dimension,^[^ ^30^ ^]^ we simulated that MTA1 directly interacts with complex I and complex V and verified that MTA1 promotes the activity of mitochondrial respiratory chain complex V, thereby supporting various energy metabolism‐related malignant behaviors of colon cancer. With the growing evidence, mitochondrion is regarded as an emerging pharmacological target in cancer.^[^ ^31^ ^]^ Targeting mitochondrion will quench the high energy demand for proliferation and metastasis phenotypes of the tumors that are vulnerable to OXPHOS inhibition.

MTA1, a member of the MTA family, has a molecular weight of 80 kDa, and is upregulated during tumor metastasis.^[^ ^32^ ^]^ MTA1 is a key component of NuRD^[^ ^33^ ^]^ and regulates nucleosome remodeling and deacetylation by stabilizing the NuRD complex and related proteins.^[^ ^34^ ^]^ MTA1 is a stronger mediator to promote cancer metastasis, such as breast cancer, colon cancer, and prostate cancer.^[^ ^32^, ^35^ ^]^ MTA1 has also been reported as a potential anticancer target, and interestingly, can even be targeted by nutrient intervention such as dietary stilbene.^[^ ^36^ ^]^ In our previous studies, we found that MTA1 is localized in the nucleus in most mouse tissues but that it is highly expressed in the cytoplasm in mouse cardiac and skeletal muscle. In most developing tissues, such as the brain, eyes, liver and intestines, of mouse embryos, MTA1 staining is mainly observed in the cytoplasm.^[^ ^15a^ ^]^ Therefore, we speculate that cytoplasmic MTA1 is more highly expressed in energy‐consuming and metabolically active tissues during embryogenesis. Muscle, the brain, and the liver also require large amounts of energy for their physiological functions. Consistent with previous findings and hypotheses, we found that MTA1 plays an important role in energy metabolism in a mitochondria‐dependent manner.

Colon cancer liver metastasis is accompanied by metabolic reprogramming of the hepatic microenvironment.^[^ ^37^ ^]^ Metastatic colon cancer cells also show metabolic dysregulation.^[^ ^38^ ^]^ Despite the advancements that have been made in colon cancer treatment,^[^ ^3^, ^39^ ^]^ much work is still needed to identify new biomarkers and therapies to improve the survival of metastatic colon cancer. In this study, it was found that MTA1 levels are positively correlated with ATP5A levels and that MTA1 promotes mitochondrial energy production, thereby regulating colon cancer metastasis. MTA1 is a potential biomarker for evaluating the metastatic potential of colon cancer and predicting the sensitivity of tumors to certain metabolism‐associated drugs.

AMPK is a major regulator of cellular energy homeostasis that coordinates multiple metabolic pathways to balance energy supply and modulate cellular growth.^[^ ^40^ ^]^ AMPK directly binds ATP, ADP, and AMP and is activated by decreased ATP generation (conversion of AMP and ADP to ATP).^[^ ^41^ ^]^ mTOR signaling is inhibited by activated AMPK via multiple mechanisms.^[^ ^40^ ^]^ There are many important mTOR‐related pathways involved in CRC, including the Wnt, PI3K/AKT, p53, and RAS/RAF/mitogen‐activated protein kinase pathways.^[^ ^42^ ^]^ In this study, we found that MTA1‐KO enhanced the sensitivity of metastatic colon cancer to everolimus and rapamycin in vitro and in vivo. Rapamycin, an mTOR inhibitor, was approved to treat metastatic renal cell carcinoma and breast cancer patients.^[^ ^21^ ^]^ Everolimus, another mTOR inhibitor, was found to have only a moderate therapeutic effect on colon cancer in multiple clinical trials, but when used in combination with bevacizumab, it was found to prolong progression‐free survival in patients with metastatic colon cancer.^[^ ^42^ ^]^ Here, we used rapamycin and everolimus to evaluate the MTA1‐ATP5A‐mediated AMPK/mTOR pathway in cancer treatment. These drugs are clinically available and are ideal targets to be translated in clinical usage. We further confirmed that the level of MTA1 was associated with the response of breast cancer patients to mTOR inhibitors. Thus, MTA1 may be a potential biomarker and therapeutic target for everolimus‐resistant cancer.

Targeting mitochondria as cancer treatment is an emerging novel strategy. Many strategies targeting mitochondrial glucose metabolism were reviewed by Ali F et al. and Roth KG et al.^[^ ^31^, ^43^ ^]^ To advance these strategies into clinic, there are some points to be considered, such as how to combinedly suppress OXPHOS and glycolysis in the tumor cells but leave the normal cells unaffected and how to exquisitely identify the tumors highly dependent on ATP from OXPHOS at the genetic and epigenetic levels.^[^ ^44^ ^]^ With these aims, the value of MTA1 should be further evaluated. This field is on fast development and expected to see great advancement soon.

Overall, this study revealed that the interaction between mitochondrial MTA1 and ATP5A drives cancer liver metastasis by increasing OXOPHOS and ATP production. Though there are still some topics that need to be clarified in the future such as how MTA1 regulates glycogen storage and motivation and which interaction domain of MTA1 is the most important domain for regulating ATP production, this research found new regulatory component to the essential “energy powerhouse” in the cell‐ATP synthase complex and discovered new mechanisms through which colon cancer liver metastasis is mediated and “Warburg effect” is refined to accommodate cancer adaptation to tumor microenvironment. These results support future assessment of MTA1‐mediated mitochondrial glucose metabolism reprogramming as the therapeutic target against cancer.

## 4 Experimental Section

STAR methods are discussed here.

### Cell Culture

The HCT116, HCT8, CT26, MCF7, MDA‐MB‐231, and 293T cell lines were obtained from the National Infrastructure of Cell Line Resources (Beijing, China). All the cell lines used in this study were cultured in DMEM (Gibco) supplemented with 10% fetal bovine serum (FBS) (HyClone) and 1% penicillin/streptomycin (HyClone) at 37 °C in a humidified incubator containing 5% CO_2_. All the cells underwent short tandem repeat (STR) profiling for authentication. MTA1‐KO HCT116 and HCT8 cell lines were generated by our group previously.^[^ ^15b^ ^]^ Briefly, CRISPR‐cas9 was used to knock out MTA1 in the human HCT116 and HCT8 colon cancer cell lines. The two pairs of single guide RNA (sgRNA) sequences for human MTA1 were designed by using the design tool from Feng Zhang Lab. The lentiviruses for the MTA1 sgRNAs or vector control were generated in HEK293T cells by cotransfection of LentiCRISPRv2 (a gift from Feng Zhang; Addgene plasmid#52961) with the packaging vectors pMD2G and psPAX2 using lipofectamine 2000 (Invitrogen). The HCT116 cells and HCT8 cells were infected with lentivirus for 72 h and were then selected with 2 µg mL^−1^ puromycin. The MTA1 protein level was assessed 2 weeks after the puromycin selection. Cells were tested for mycoplasma prior to the experiment, and mycoplasma was tested by MycoCheck Mycoplasma PCR Detection Kit (A058‐1, ABP Biosciences).

### Real‐Time Proliferation Assay

Cell growth was measured as described previously.^[^ ^13^ ^]^ Proliferation was measured by xCELLigence Real‐Time Cell Analysis (RTCA) (ACEA Biosciences, San Diego). Cells were seeded in 16‐well E‐plates, and the cell index was recorded every hour for 72 h. The MTA1‐OE and control HCT116 cells were treated with oligomycin at a final concentration of 2.5 mm. The cell index was normalized to that of control cells (normalized cell index). Experiments were performed in triplicate; the results are presented as the mean ± SD.

### Colony Formation Assay

The colony formation assay was performed as described previously.^[^ ^13^ ^]^ Briefly, cells were seeded in 60 mm dishes at a density of 2 × 10^3^ cells per well. Three days later, the cells were treated with oligomycin at a final concentration of 2 µm for 10 days. Medium with or without the inhibitor was replaced every 3 days. When the colonies formed by control cells were ≈ 50 cells per colony, the cells were fixed with methyl alcohol for 15 min; and then, stained with a 0.5% crystal violet solution for 15 min. The colonies were counted by a gel documentation system (G:BOX F3, Syngene, USA) and analyzed. Experiments were performed in triplicate and the results are presented as the mean ± SD.

### Gene Expression Analysis by qPCR

Total RNA was isolated from HCT116 and HCT8 cells using TRIzol reagent. Then, 1 mg of RNA was treated with genomic DNase and reverse transcribed using a PrimeScript RT Reagent Kit (RR037A, Takara). qPCR was performed in triplicate with 25 ng of cDNA using validated qPCR primers, a TB Green Premix Ex Taq II Kit (RR820A, Takara), and the Applied Biosystems Real‐Time PCR System (QSDX, Thermo Fisher, USA) as previously described.^[^ ^45^ ^]^ The following primers were used for qPCR:

PYGL: FWD 5′‐CAGCCTATGGATACGGCATTC‐3′; REV 5′‐

CGGTGTTGGTGTGTTCTACTTT‐3′

PYGM: FWD 5′‐CCATGCCCTACGATACGCC‐3′; REV 5′‐

TAGCCACCGACATTGAAGTCC‐3′

GSK3A: FWD 5′‐CACAGTCGTAGCCACTCTAGG‐3′; REV 5′‐

GTCCAGCTTACGCATGATCTG‐3′

GSK3B: FWD 5′‐GGCAGCATGAAAGTTAGCAGA‐3′; REV 5′‐

GGCGACCAGTTCTCCTGAATC‐3′

GYS1: FWD 5′‐GCGCTCACGTCTTCACTACTG‐3′; REV 5′‐

TCCAGATGCCCATAAAAATGGC‐3′

GBE1: FWD 5′‐GGAGATCGACCCGTACTTGAA‐3′; REV 5′‐

ACATCTGTGGACGCCAAATGA‐3′

PGM1: FWD 5′‐ CCAAACCGACTGAAGATCCGT‐3′; REV 5′‐

CATGTTTCGATCCCCATCTCC‐3′

UGP2: FWD 5′‐ATGTCTCAAGATGGTGCTTCTCA‐3′; REV 5′‐

GGTGTGCTCAAATTCATGTGATG‐3′

HK2: FWD 5′‐GAGCCACCACTCACCCTACT‐3′; REV 5′‐

CCAGGCATTCGGCAATGTG−3′

LDHA: FWD 5′‐ATGGCAACTCTAAAGGATCAGC‐3′; REV 5′‐

CCAACCCCAACAACTGTAATCT−3′

PFKL: FWD 5′‐GCTGGGCGGCACTATCATT‐3′; REV 5′‐

TCAGGTGCGAGTAGGTCCG−3′

MDH1: FWD 5′‐GGTGCAGCCTTAGATAAATACGC‐3′; REV 5′‐

AGTCAAGCAACTGAAGTTCTCC−3′

SCO2: FWD 5′‐ACAGGCTCTCTCAGCTCAAG−3′; REV 5′‐

CAGGGCTTCTGTTCGCTTTTG−3′

G6PD: FWD 5′‐CGAGGCCGTCACCAAGAAC−3′; REV 5′‐

GTAGTGGTCGATGCGGTAGA−3′

PKLR: FWD 5′‐GGACACGGCATCAAGATCATC‐3′; REV 5′‐

GCAGCGCCCAATCATCATC‐3′

The reaction was performed using the QSDX Real‐Time PCR System according to the manufacturer's protocol. The data were analyzed by the 2−^∆∆Ct^ method. Experiments were performed in triplicate.

### In Vitro Invasion Assay

To evaluate the invasion capacity of the cells, 2 × 10^5^ MTA1‐OE cells were seeded in the upper chamber of a 24‐well Transwell plate precoated with 0.5% Matrigel in growth medium without serum. The bottom chambers of the 24‐well plate were filled with 600 µL DMEM containing 20% FBS, and the Transwell plates were incubated at 37 °C in a humidified incubator containing 5% CO_2_ for 48 h. The cells were treated with oligomycin at a final concentration of 2 µm. The cells were then fixed in methanol for 15 min and stained with 0.5% crystal violet for 15 min, and the cells on the upper surface that did not pass through the membrane were removed with a cotton swab. The cells on the lower surface of the chamber membrane were photographed. Seven random microscopic fields were selected, and the average area occupied by cells was calculated with ImageJ software. Experiments were performed in triplicate and the results are presented as the mean ± SD.

### Mitochondrial Glucose Metabolism Assay

To evaluate the balance between mitochondrial OXPHOS and glycolysis, the oxygen consumption rate (OCR) and extracellular acidification rate (ECAR) of HCT116 and HCT8 cell sublines were measured using a Seahorse XF96 Extracellular Flux Analyzer (Seahorse Bioscience, North Billerica, USA) as previously described.^[^ ^46^ ^]^ Cells were seeded at an appropriate density (12 000 cells for HCT116; 8000 cells for HCT8) in XFe96 FluxPaks (102416‐100, Seahorse) and incubated overnight in growth medium. 18 h later, the medium was replaced with bicarbonate‐free low‐buffered assay medium containing 2 mm glutamine (G8540, Sigma) for ECAR measurement or 25 mm glucose (G8769, Sigma), 2 mm glutamine, and 1 mm pyruvate (S8636, Sigma) for OCR measurement at pH ≈ 7.4, and the cells were incubated for 60 min at 37 °C in a CO_2_‐free incubator. A Seahorse XF Cell Mito Stress Test Kit (103015‐100, Seahorse) and Seahorse XF Glycolysis Stress Test Kit (S7805A, Seahorse) were used to assess mitochondrial stress and glycolysis stress, respectively. For the mitochondrial stress test, oligomycin, FCCP, and rotenone were injected by the instrument from the reagent ports into the XFe96 FluxPaks wells at final concentrations of 2, 1, and 5 µm, respectively. For the glycolysis stress test, the final concentrations of glucose, oligomycin, and 2‐deoxyglucose were adjusted to 10 mm, 2 µm, and 50 mm, respectively.

### ATP, Glycogen, and Reactive Oxygen Species (ROS) Assays

The level of ATP in the cells was measured using an ATP assay kit (S0026, Beyotime). Briefly, 2  × 10^6^ cells per well were seeded in 60 mm dishes. After overnight culture, the cells were lysed with ATP lysis buffer. The supernatants were collected after centrifugation at 12 000 rpm for 10 min at 4 °C, and the signal was detected using a luminometer (H1, BIOTEK). Total protein lysates were extracted, and the protein concentration was measured using the Bradford Protein Assay Kit (Detergent Compatible) (P0006C, Beyotime) for normalization. The glycogen level was measured using a Glycogen Colorimetric/Fluorometric Assay Kit (K646‐100, BioVision). In this assay, glucoamylase hydrolyzes glycogen to glucose, which is then specifically oxidized to produce a product that reacts with the OxiRed probe to generate a detectable color (OD570 nm). The reaction samples were analyzed using a multiwell spectrophotometer (H1, BIOTEK). Both of these experiments were repeated three times. Intracellular ROS levels were measured using a Reactive Oxygen Species Assay Kit (S0033, Beyotime). Briefly, 2  × 10^5^ cells per well were seeded in 6‐well plates. After overnight culture, the cells were incubated with DFCH‐DA at a final concentration of 10 µm at 37  °C for 20 min and then analyzed by flow cytometry (LSRII, BD). The MMP was measured using an MMP assay kit with JC‐1 (C2006, Beyotime). Briefly, 2  × 10^5^ cells per well were seeded in 6‐well plates. After overnight culture, the cells were incubated with 1X JC‐1 working solution at 37  °C for 20 min, washed twice with 1X JC‐1 staining buffer, and then analyzed by flow cytometry (LSRII, BD). All experiments were performed in triplicate.

### ATP Synthase Specific Activity Assay

An ATP Synthase Specific Activity Microplate Assay Kit (ab109716, Abcam) was used to assess both the activity and level of ATP synthase as previously described.^[^ ^47^ ^]^ Briefly, 50 µg of protein lysate from various cell samples was added to the wells of a microplate, and the enzyme was immunocaptured on the surface of the wells by precoated antibodies. ATP synthase activity was measured by determining the rate of ATP hydrolysis to ADP, and the ultimate production of ADP, which is coupled to the oxidation of NADH to NAD+, was assessed by measuring the decrease in absorbance at 340 nm. In these same wells, the quantity of ATP synthase was measured by adding an ATP synthase‐specific antibody conjugated to alkaline phosphatase. The level of ATP synthase was measured and is presented as the change in the absorbance at 405 nm.

### Coimmunoprecipitation (Co‐IP)

To evaluate the physical interaction between MTA1 and potential target proteins, HCT116 and HCT8 cells were lysed in NP‐40 lysis buffer (50 mm Tris‐HCl, pH 8.0, 0.5% NP‐40, 10% glycerol, 150 mm NaCl, 2 mm MgCl_2_, and 1 mm EDTA; supplemented with protease inhibitors). A total of 1 mg cell lysate was incubated with 2 µg a rabbit polyclonal MTA1 primary antibody (ab71153, Abcam) overnight at 4 °C on a rotating shaker followed by 20 µL protein A/G beads (sc2003, Santa Cruz) for 6 h at 4 °C. The immunoprecipitants were washed six times with NP‐40 buffer and collected after centrifugation at 3500 × *g* for 5 min. Subsequently, the beads were boiled in SDS loading buffer for 10 min, and the samples were subjected to Western blotting. All experiments were performed in triplicate.

### Immunofluorescence

MTA1 and ATP5A colocalization were assessed using immunofluorescence. Briefly, cells were cultured on sterilized coverslips. MitoTracker Deep Red FM (#8778, Cell Signaling Technology) was added to the growth medium at a final concentration of 500 nm, and the cells were incubated for 30 min at 37 °C. After incubation, the cells were fixed with 4% paraformaldehyde at room temperature for 15 min, rinsed with PBS three times for 5 min each, permeabilized with 0.25% Triton X‐100 at room temperature for 10 min; and then, blocked with 0.5% bovine serum albumin for 30 min. The coverslips with cells were subsequently incubated overnight with a mouse monoclonal MTA1 primary antibody (ab51266, Abcam) and rabbit antibody against ATP5A (14676‐1‐AP, Proteintech); and then, incubated with corresponding fluorescence‐conjugated secondary antibodies diluted in blocking buffer for 1 h. The coverslips with cells were finally mounted with mounting medium containing DAPI. Images were acquired using confocal laser scanning microscopy (GE, DeltaVision OMX V4/SR).

### Identification of MTA1‐Related Metabolites by Mass Spectrometry

MTA1‐KO and control HCT116 cells were collected in 1.5 mL centrifuge tubes. Then, 1 mL precooled extractant (70% methanol aqueous solution) was added to the tubes, and the tubes were agitated for 1 min. Then, the mixture was frozen in liquid nitrogen for 3 min and the cells were agitated three times for 2 min each. The mixture was centrifuged again at 12 000 rpm and 4 °C for 10 min; and then, the supernatants were transferred to sample bottles for LC–ESI–MS/MS (UPLC, Shim‐pack UFLC SHIMADZU CBM A system; MS, QTRAP 6500+ System).

### Analysis of MTA1‐Related Liver Metastasis of Colon Cancer In Vivo

All animal studies were conducted according to the guidelines of the Animal Control Committee of National Cancer Center/National Clinical Research Center for Cancer/Cancer Hospital, the Chinese Academy of Medical Sciences, and the Peking Union Medical College. CRC liver metastasis mouse model was created by intrasplenic injection of HCT116 cells, as previously described.^[^ ^48^ ^]^ Six‐week‐old male BALB/c‐Nu mice were randomly divided into eight groups (*n* = 5 per group), and cells (4.0 × 10^6^/50 µL) were slowly injected into the spleen of BALB/c‐nu/nu mice with an insulin syringe and the blood was compressed after 3 min of hemostasis. The mice were injected intraperitoneally with 100 µL of 8.8 mg mL^−1^ rapamycin every other day 2 weeks after the spleen‐tail injection. The body weight of each mouse was measured every 3 days. The mice were sacrificed on day 25 according to data from pilot experiments. The experiments were conducted in blinded manner and the animals were randomized to the treatment groups for the drug treatments. The livers were isolated, weighed, photographed; and then, analyzed by serial hematoxylin & eosin stain and the liver metastatic foci were counted.

### Computational Simulation of the Interaction Between MTA1 and the ATP Synthase Complex

MOPAC2016,^[^ ^18^ ^]^ including the PM7^[^ ^49^ ^]^ (semiempirical Hamiltonian) method, MOZYME^[^ ^50^ ^]^ (a localized molecular orbital method that replaces the standard SCF procedure for easier calculations of large organic compounds), the ADDH process (adds hydrogen atoms to ATP synthase), and the solvation process^[^ ^51^ ^]^ (approximates the effect of a solvent model surrounding the molecule, such as the stabilization of ions on the surface of a protein), were employed to study the interaction between ATP synthase and the MTA1 protein. The amino acid sequence of MTA1 was obtained from the UniProt database, and its protein code was Q13330. The current crystal structure of MTA1 was still incomplete; so, the amino acid fragments included in the simulation were amino acids 162‐354, 464‐546, and 656‐711. MOPAC uses a large unit cell called a “cluster” and adopts the Born–von Kármán periodic boundary conditions. Dynamic reaction coordinate (DRC) simulation, a molecular dynamics (MD) method, was employed to investigate the interaction between ATP synthase and anesthetic molecules.^[^ ^52^ ^]^ The simulation temperature was set to 300 K, and the simulation lasted for 2 ps, with a 1 fs interval and a total of 2000 steps. The “half‐life” for the loss of kinetic energy was 50 fs. The binding energies (Δ*E*_bind_) of MTA1 fragments were investigated to evaluate the binding strength between them and ATP synthases, which was calculated by the following formula:

(1) $$\Delta E_{\mathrm{bind}}=E_{\mathrm{composite}}-E_{M}-E_{\mathrm{enzyme}}$$

where *E*_M_ indicates the total energy of the MTA1 fragment, *E*_composite_ indicates the total energy of the M‐enzyme composite, and *E*_enzyme_ indicates the part of ATP synthase around the binding sites. A more negative Δ*E_bind_* indicates that the heat of formation is greater and the interaction between MTA1 and ATP synthase is stronger.

### Evaluation of the Association of MTA1 With Survival and Treatment Sensitivity in Clinical Cancer Specimens

The present study was approved by the Ethics Committee of Cancer Hospital, Chinese Academy of Medical Sciences, and Peking Union Medical College. A total of 180 human colon cancer specimens and adjacent specimens were collected from August 2012 to July 2016 at the Cancer Hospital, Chinese Academy of Medical Sciences and Peking Union Medical College. Fifty‐eight human breast cancer specimens, including breast tissues obtained during surgery and breast or liver biopsies from metastatic tumors, were collected from May 2012 to November 2019 at the Cancer Hospital, Chinese Academy of Medical Sciences and Peking Union Medical College. These cancer specimens were examined and a diagnosis was made by pathologists at the Cancer Hospital, Chinese Academy of Medical Sciences and Peking Union Medical College. Informed consent was obtained from all patients who provided samples.

### Immunohistochemical (IHC) Staining and Scoring of Cancer Tissues from Patients and Animals

Tissue microarrays (TMAs) were constructed by harvesting 400 µm tissue cores from paraffin‐wax embedded samples collected from 180 CRC patients. The TMAs were heated at 60 °C overnight, deparaffinized in xylene for 30 min, and rehydrated with gradient alcohol solutions. Then, the TMAs were boiled in EDTA antigen retrieval solution (ZLI‐9066, Zsgb‐Bio, Beijing, China) for 30 min, treated with an endogenous peroxidase blocker (PV‐9000, Zsgb‐Bio) for 10 min to block endogenous peroxidase activity, and incubated with normal goat serum (ZLI‐9022, Zsgb‐Bio) for 10 min. Next, the slides were incubated overnight with rabbit monoclonal primary antibodies against ATP5A (14676‐1‐AP, Proteintech), mTOR (#2972, Cell Signaling Technology), and phosphorylated mTOR (#2971, Cell Signaling Technology) at 4 °C. The next day, the slides were incubated for 25 min with polymerase adjuvant and biotinylated secondary antibodies (PV‐9000, Zsgb‐Bio), and color development was performed with DAB (ZLI‐9019, Zsgb‐Bio). Last, the slides were counterstained with hematoxylin and mounted with neutral balsam. IHC staining of the tissue was evaluated in a double‐blinded manner by using a semiquantitative immunoreactivity scoring system (IRS) for both staining intensity (0: negative; 1: weak; 2: moderate; 3: intense) and percentage of positively stained cancer cells (0: none; 1: <10%; 2: 10−50%; 3: 51−80%; and 4: >80%). The final staining scores ranged from 0 to 12, with a score >9 indicating strong expression, a score >4 and ≤8 representing moderate expression, a score ≥1 and ≤4 indicating weak expression, and a score of 0 representing no expression.

### Bioinformatics Functional Enrichment Analysis of Genes

To analyze the functions of genes, the DAVID Bioinformatics Resources 6.8 (https://david.ncifcrf.gov/), GeneOntology (http://www.geneontology.org/), and STRING (https://cn.string‐db.org/) databases were used for enrichment analysis. Fisher's exact test was used to determine the degree of enrichment.

### Statistical Analysis

Statistical analyses and generation of graphics were performed using GraphPad Prism 7 and IBM SPSS Statistics 26. All data were expressed as mean ± SEM. For the statistic results of immunofluorescence and in vivo experiments data, if only two groups were applied, Student's *t*‐test was used; if more than three groups were applied, one‐way ANOVA was used. For the OCR, ECAR, RTCA, colony formation, and invasion assay data, two‐way ANOVA was used. If the differences were significant, Tukey's multiple comparisons test was applied to compare values at different time points. Details of statistical tests used are indicated in the respective figure legends. *P* value < 0.05 (two‐tailed) was considered statistically significant, with *p < 0.05, **p < 0.01, ***p < 0.001, and ****p < 0.0001, respectively.

### Ethics Approval and Consent to Participate

The collection and use of colon cancer tissues and breast cancer tissue slide were approved by the Institutional Review Board of National Cancer Center/National Clinical Research Center for Cancer/Cancer Hospital, Chinese Academy of Medical Sciences and Peking Union Medical College (NCC2018‐047). All animal studies were approved by the Animal Control Committee of the National Cancer Center/National Clinical Research Center for Cancer/Cancer Hospital, Chinese Academy of Medical Sciences, and Peking Union Medical College (ID: NCC2018A017).

## Conflict of Interest

The authors declare no conflict of interest.

## Author Contributions

T.W., F.S., C.L, P.N., and Y.S. contributed equally to this work. T.W., F.S., C.L., P.N., J.W., and Y.Z. contributed to manuscript writing, experiment performance, and data analysis. Y.S., H.M., A.E.H. and D.X. contributed to cancer tissues collection and data analysis. Y.G. and X.W. contributed to computational simulation and analysis. H.Q., F.M., and Q.Z. contributed to study design and supervision. All authors have read and approved this manuscript and agree to its submission to this journal.

## Supporting information

Supporting Information

Supplemental Table 2

## Data Availability

The data that support the findings of this study are available from the corresponding author upon reasonable request.
