# Supplementary material for: MTA1, a Novel ATP Synthase Complex Modulator, Enhances Colon Cancer Liver Metastasis by Driving Mitochondrial Metabolism Reprogramming
Source: Adv Sci (Weinh). 2023 Jul 13;10(25):2300756. doi: 10.1002/advs.202300756 (PMC10477900; doi:10.1002/advs.202300756)
Supplement: Supplementary file 1 — Supporting Information [file ADVS-10-2300756-s002.pdf]

ADVANCED SCIENCE
Open Access

Supporting Information

for *Adv. Sci.*, DOI 10.1002/advs.202300756

MTA1, a Novel ATP Synthase Complex Modulator, Enhances Colon Cancer Liver Metastasis by Driving Mitochondrial Metabolism Reprogramming

Ting Wang, Fangzhou Sun, Chunxiao Li, Peng Nan, Yan Song, Xuhao Wan, Hongnan Mo, Jinsong Wang, Yantong Zhou, Yuzheng Guo, Aya Ei Helali, Dongkui Xu, Qimin Zhan*, Fei Ma* and Haili Qian**

MTA1, a novel ATP synthase complex modulator, enhances colon cancer liver metastasis by driving mitochondrial metabolism reprogramming

Ting Wang, Fangzhou Sun, et al…

State Key Laboratory of Molecular Oncology, National Cancer Center/National Clinical Research Center for Cancer/Cancer Hospital, Chinese Academy of Medical Sciences and Peking Union Medical College, Beijing, China

Supplementary Figures

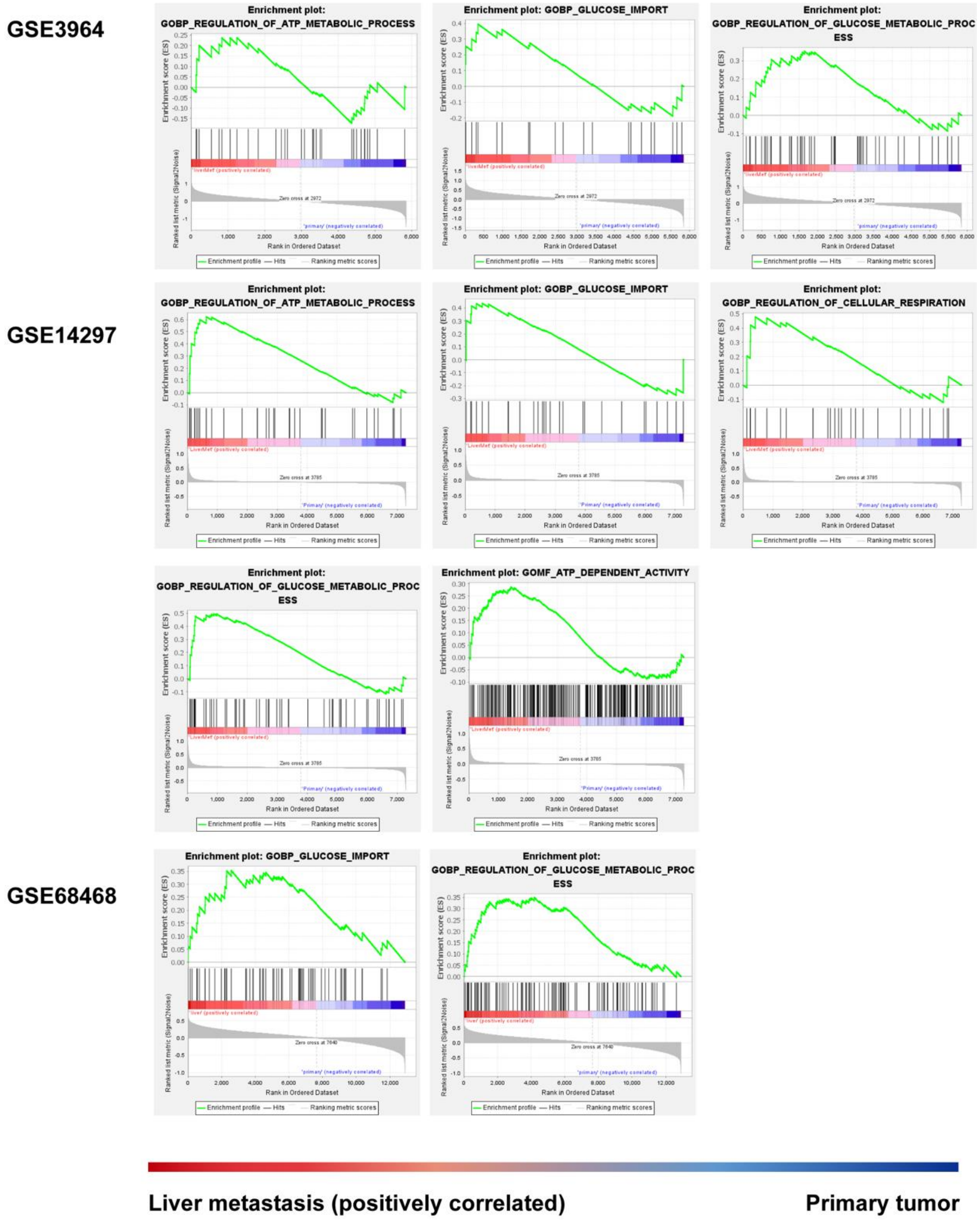

Figure S1 GSEA showed expression of genes in the liver metastases which were upregulated compared with primary CRC was positively correlated with glucose import, glucose metabolism process and ATP metabolism process.

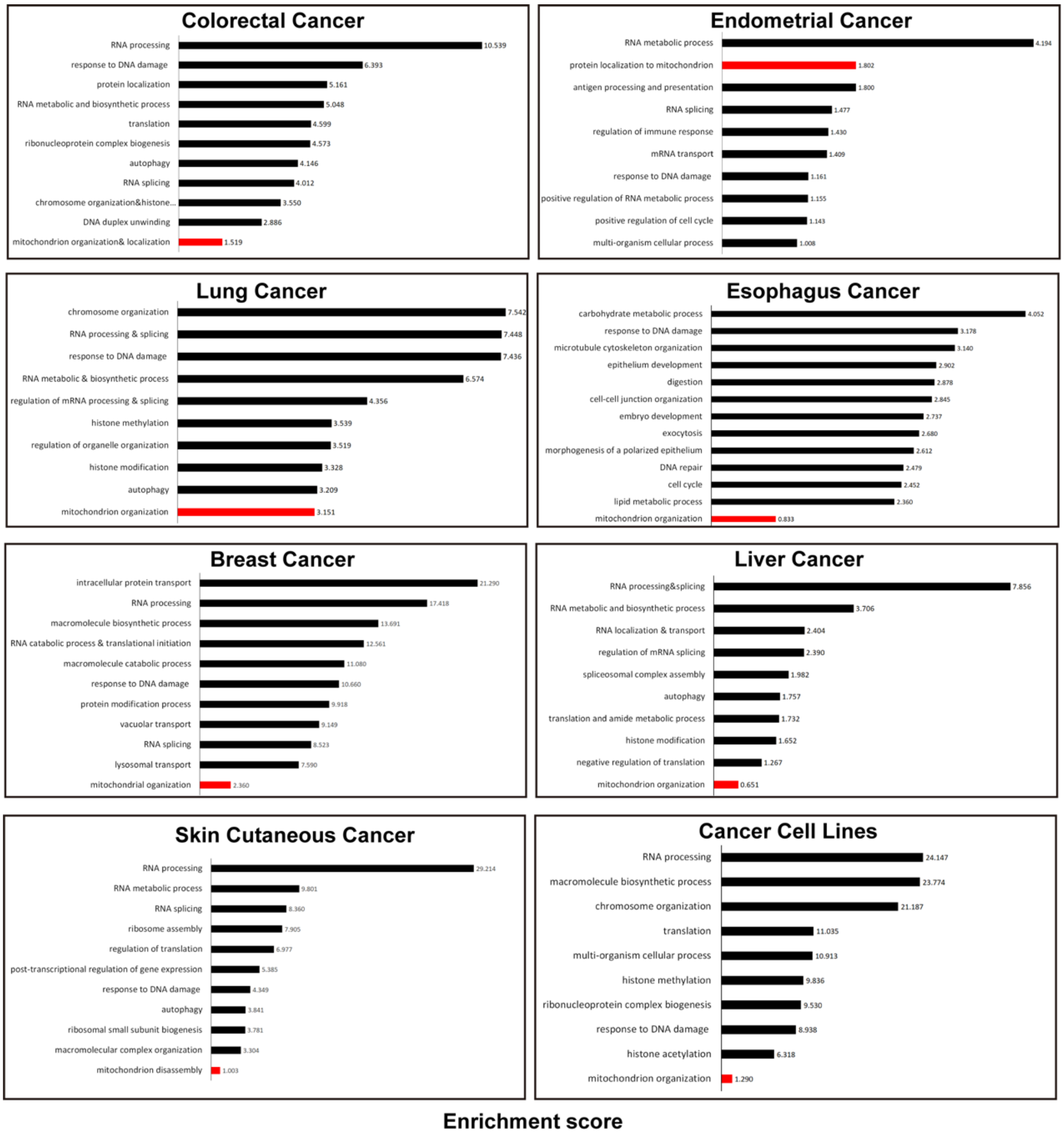

Figure S2 Functional enrichment analysis of genes co-expressed with MTA1 according to pan-cancer data from the TCGA using the DAVID 6.8 database.

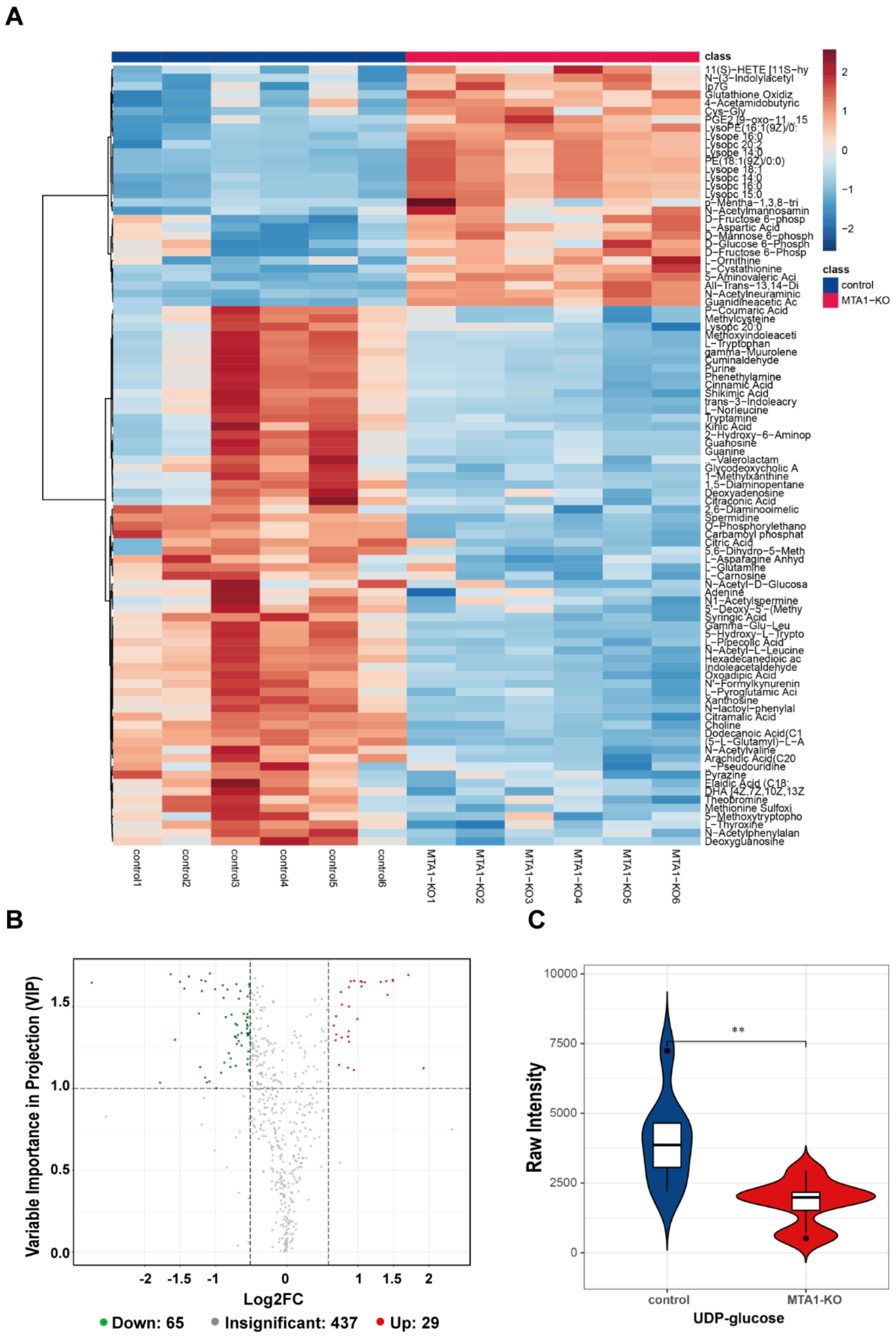

Figure S3 (A-B) Heatmap (A) and volcano plot (B) of 94 differentially expressed metabolites in MTA1-KO HCT116 cells versus control cells. (C) Violin plot of the expression of the differentially expressed metabolite UDP-G in MTA1-KO HCT116 cells versus control cells; n=6 replicate/group. The values are the mean ± SD (**p < 0.01).

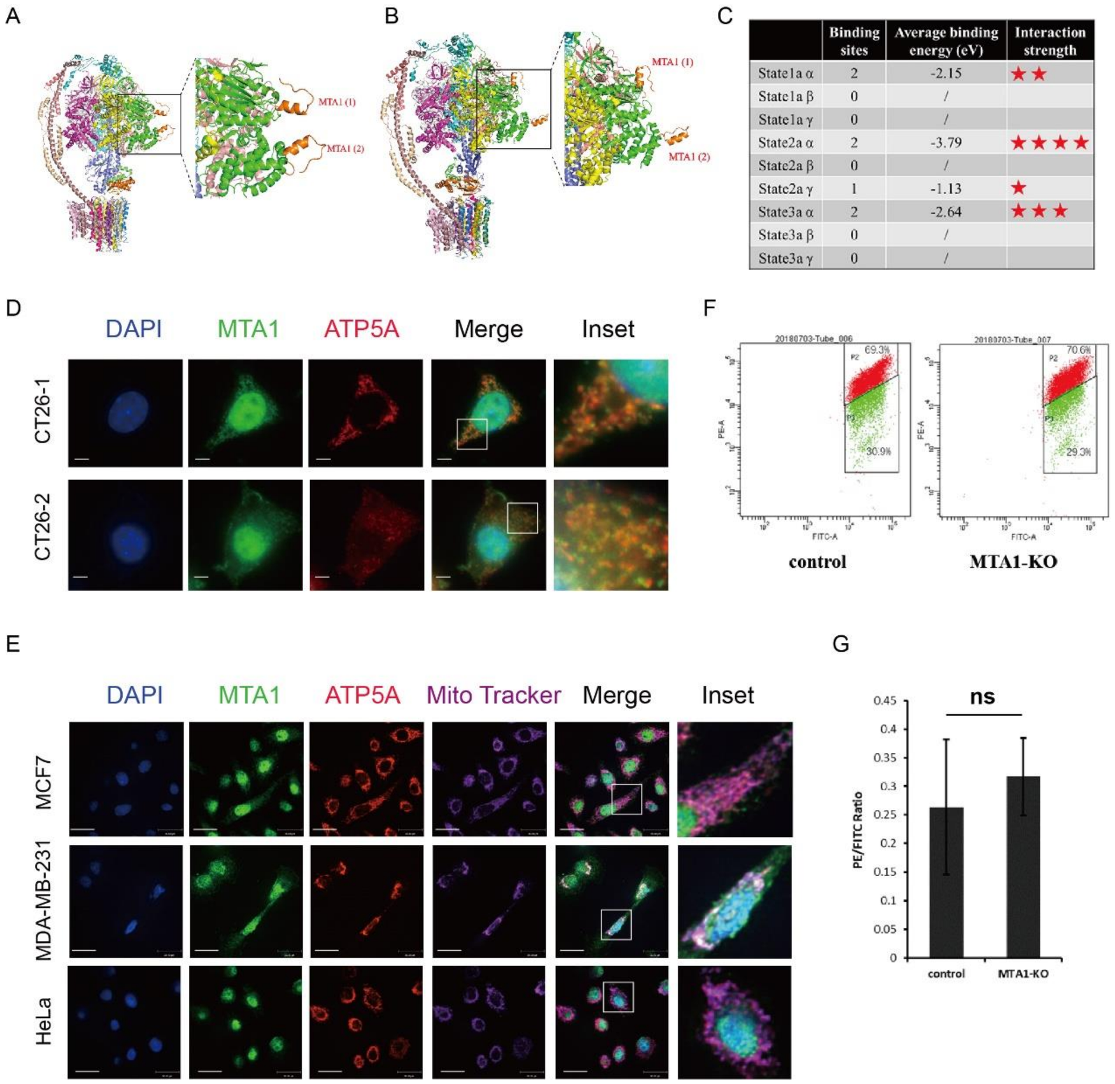

	Binding sites	Average binding energy (eV)	Interaction strength
State1a α	2	-2.15	★★
State1a β	0	/	
State1a γ	0	/	
State2a α	2	-3.79	★★★★
State2a β	0	/	
State2a γ	1	-1.13	★
State3a α	2	-2.64	★★★
State3a β	0	/	
State3a γ	0	/	

Figure S4 (A-B) Simulation of the interaction between amino acids 670-695 of MTA1 and ATP synthase in state 1 (A) and state 3 (B). Binding sites are indicated by black boxes and magnified. MTA1 peptides are shown in orange (top). The ATP synthase α subunit is shown in green. (C) Table of the binding site number of the MTA1 peptide on each ATP synthase subunit and the interaction strength. (D) Colocalization of MTA1 and ATP5A in mouse derived cancer cells CT26. Scale bar, 5 μm. (E) Colocalization of MTA1 and ATP5A in mitochondria in MCF7, MDA-MB-231 and HeLa cells. Scale bar, 30 μm. (F-G) MMP of MTA1-KO and control cells. The data are representative of two biological replicates. The values are the mean ± SD (ns, not significant).

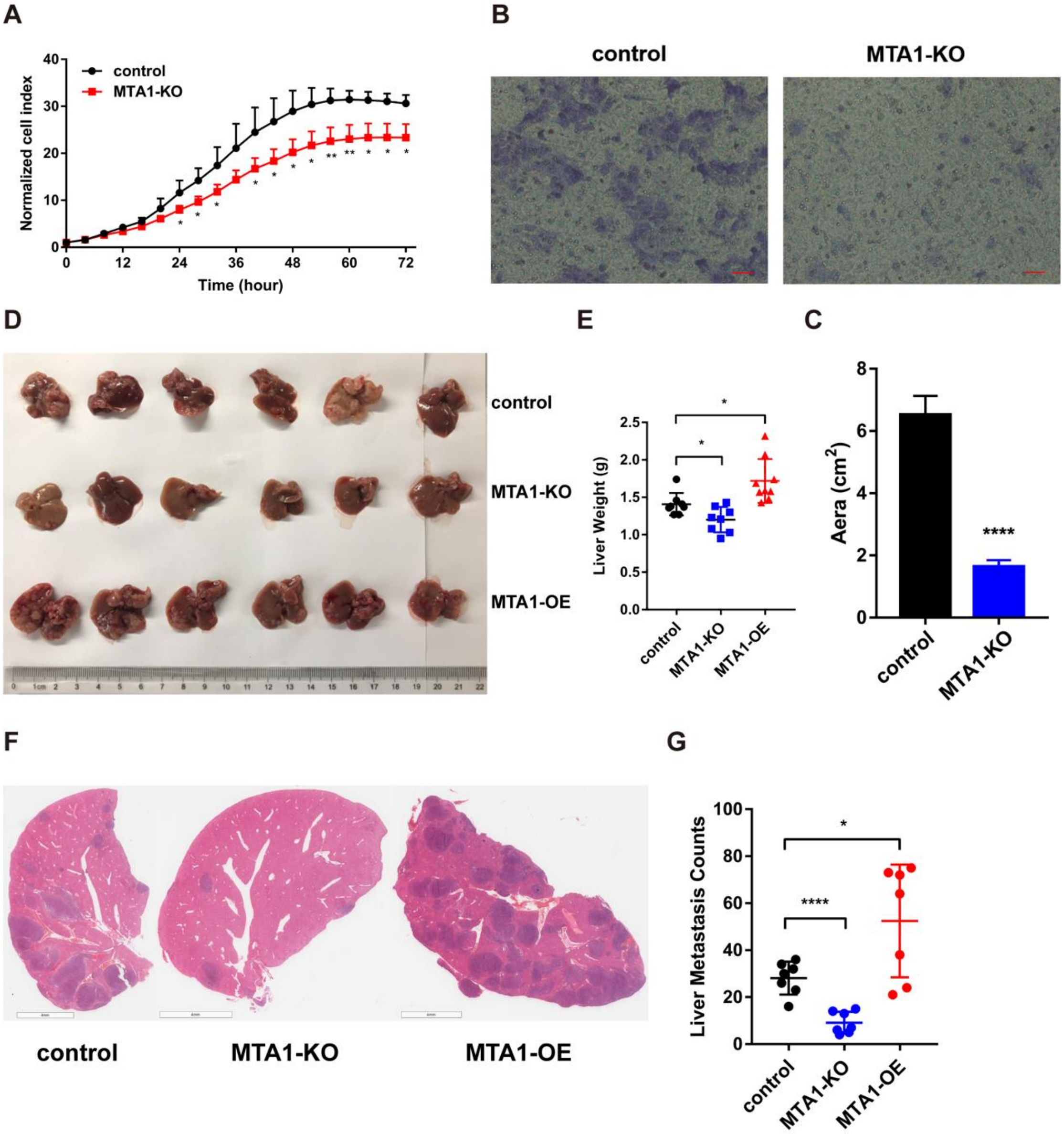

Figure S5 (A) The proliferation of MTA1-KO and control HCT116 cells. The values are the mean ± SEM (**p < 0.01, *p < 0.05; ns, not significant). (B-C) The invasion of MTA1-KO and control HCT116 cells cultured in Transwell plates for 48 hours. The values are the mean ± SD (****p < 0.0001), , n=3 replicate/group. Scale bar, 100 μm. (D) Representative image of the liver metastatic burden in Balb/c-nu/nu mice injected with MTA1-KO, MTA1-OE and control cells via the spleen tail; n=6 mice/group. (E) Liver weight of the mice with liver metastasis shown in (D). The values are the mean ± SD (*p < 0.05; ns, not significant). (F-G) Representative HE staining images (F) and number (G) of the liver metastases (****p < 0.0001, *p < 0.05).

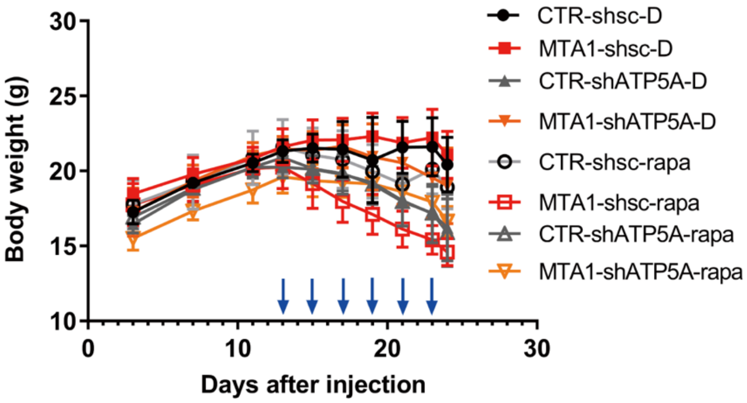

Figure S6 The body weight of mice with liver metastasis in the in vivo experiment. The blue arrow shows the day of rapamycin intraperitoneal injection.

Supplementary Tables

Table S1. 27 mitochondria-associated proteins captured by rabbit- and mouse-derived MTA1 antibodies

No.	Protein	No.	Protein
1	MT-ATP6	15	HSPA9
2	ATP5O	16	HSPD1
3	ATP5A	17	IMMT
4	ATP5B	18	LRPPRC
5	ATP5C1	19	LRRC59
6	ATP5H	20	MFF
7	ATAD3A	21	MRPL11
8	ATAD3B	22	PHB2
9	BRI3BP	23	PHB
10	DDX3X	24	RPS3
11	DHX30	25	SLC25A11
12	NDUFS1	26	SLC25A5
13	NDUFV2	27	VDAC2
14	HSPA1L		
